# A semi-automatic deep learning model based on biparametric MRI scanning strategy to predict bone metastases in newly diagnosed prostate cancer patients

**DOI:** 10.3389/fonc.2024.1298516

**Published:** 2024-06-11

**Authors:** Song Xinyang, Shen Tianci, Hu Xiangyu, Zhang Shuang, Wang Yangyang, Du Mengying, Xu Tonghui, Zhou Jingran, Yang Feng

**Affiliations:** ^1^ Department of Radiology, Xiangyang No. 1 People’s Hospital, Hubei University of Medicine, Xiangyang, China; ^2^ Department of Radiology, The People’s Hospital of Zouping City, Zouping, China; ^3^ Department of Radiology, Xiangyang Central Hospital, Affiliated Hospital of Hubei University of Arts and Science, Xiangyang, China; ^4^ Department of Orthopedics, Xiangyang No. 1 People’s Hospital, Jinzhou Medical University Union Training Base, Xiangyang, China

**Keywords:** MRI, deep learning, ResNet, bone metastasis, prostate cancer

## Abstract

**Objective:**

To develop a semi-automatic model integrating radiomics, deep learning, and clinical features for Bone Metastasis (BM) prediction in prostate cancer (PCa) patients using Biparametric MRI (bpMRI) images.

**Methods:**

A retrospective study included 414 PCa patients (BM, n=136; NO-BM, n=278) from two institutions (Center 1, n=318; Center 2, n=96) between January 2016 and December 2022. MRI scans were confirmed with BM status via PET-CT or ECT pre-treatment. Tumor areas on bpMRI images were delineated as tumor’s region of interest (ROI) using auto-delineation tumor models, evaluated with Dice similarity coefficient (DSC). Samples were auto-sketched, refined, and used to train the ResNet BM prediction model. Clinical, radiomics, and deep learning data were synthesized into the ResNet-C model, evaluated using receiver operating characteristic (ROC).

**Results:**

The auto-segmentation model achieved a DSC of 0.607. Clinical BM prediction’s internal validation had an accuracy (ACC) of 0.650 and area under the curve (AUC) of 0.713; external cohort had an ACC of 0.668 and AUC of 0.757. The deep learning model yielded an ACC of 0.875 and AUC of 0.907 for the internal, and ACC of 0.833 and AUC of 0.862 for the external cohort. The Radiomics model registered an ACC of 0.819 and AUC of 0.852 internally, and ACC of 0.885 and AUC of 0.903 externally. ResNet-C demonstrated the highest ACC of 0.902 and AUC of 0.934 for the internal, and ACC of 0.885 and AUC of 0.903 for the external cohort.

**Conclusion:**

The ResNet-C model, utilizing bpMRI scanning strategy, accurately assesses bone metastasis (BM) status in newly diagnosed prostate cancer (PCa) patients, facilitating precise treatment planning and improving patient prognoses.

## 1 Introduction

Prostate cancer (PCa) ranks as the second most common cancer in men globally (1). While endocrine therapy for PCa boasts a 5-year and 10-year survival rate of 98% and 78% (2), the onset of bone metastasis (BM) marks a decline in patient prognosis. BM can trigger a range of skeletal complications, such as hypercalcemia, pathological fractures, spinal compression, and bone pain, all of which exacerbate prognosis and heighten mortality in advanced cases (2, 3). Managing BM patients poses challenges for clinicians, with only systemic palliative care options currently available (4). Hence, precise BM prediction in PCa patients is crucial for enhancing survival quality.

Research indicates that tailored interventions for bone metastases can extend patient survival (5–9). However, the European Urological Association (EUA) guidelines classify patients with prostate-specific antigen (PSA) levels exceeding 10 ng/ml as high-risk for BM, yet their BM positive rate at follow-up is a mere 7% (10). This hints at the inadequacy of clinical features as BM risk predictors. Some retrospective studies have employed PSA-dominant clinical risk prediction models to forecast the occurrence of BM in PCa. However, the results of these studies exhibit instability (AUC=0.68–0.88) (11–16), underscoring the need for a reliable diagnostic method.

Radiomics, introduced by Lambin in 2012 (17), shows potential in predicting BM in PCa by quantitatively analyzing target regions in images. Wang, Zhang et al. first used radiomics modeling for BM prediction predictions with an attempted area under the curve (AUC) value of 0.91 (18, 19). Numerous studies have unequivocally affirmed the value of MRI in the diagnosis of prostate cancer, markedly enhancing the accuracy of PCa diagnosis (20, 21). Yet, recent evidence suggests that Dynamic Contrast Enhance (DCE) sequences may not significantly impact clinical decision-making or patient benefit (21–25). The biparametric magnetic resonance imaging (bpMRI), as an emerging scanning strategy, showcases its advantages primarily by simplifying the selection of imaging parameters while retaining sufficient information for effective medical diagnosis. It not only reduces the invasiveness and potential risks of allergic reactions to patients but also brings about higher socio-economic benefits. For patients with contraindications to contrast agents (such as renal failure), it offers more possibilities. Simultaneously, due to the alteration in imaging sequences, novel predictive models are required to adapt to this change (19). Leveraging artificial intelligence technologies such as Deep learning (DL) to autonomously derive quantitative representations from medical images is an evolving direction in radiomic research (26–28). DL technology is gaining popularity as a creative tool due to its hierarchical network structure, which possesses a high capacity for memory and the ability to analyze abstract features. This capability makes it feasible to identify tumor regions. Concurrently, transfer learning and pre-trained DL networks facilitate the execution of new tasks, enabling the application of small datasets (25–27, 29, 30). DL methods based on convolutional neural networks are an emerging approach for predicting tumor metastasis with great potential (31–35). Until now, no authoritative statement or consensus has suggested that DL is inherently superior and replaces radiomics. Therefore, the combination of DL with radiomic features may lead to exceptional performance in predicting BM.

While using the mp-MRI imaging radiomics method to differentiate between NO-BM and BM states has been examined in several articles (18, 19), the development of predictive models based on bpMRI images remains unexplored. In this study, we endeavor to distinguish BM states in PCa patients non-invasively using DL combined with Radiomics, exclusively employing bpMRI images.

## 2 Materials and methods.

### 2.1 Patient information and clinical data

This study was conducted in accordance with the Helsinki Declaration and was approved by the ethics committees of both institutions. Due to retrospective study, the requirement for patient informed consent was waived.

Center 1 collected 318 patients which, including 95 patients with BM and 223 patients with PCa without BM (Non-BM patients), divided into an internal test cohort, validation cohort, and a training cohort according to a ratio of 3:1:6 between January 2016 and December 2022. The Center 2 collected data from 96 patients between March 2018 and December 2022, including 41 BM patients and 55 Non-BM patients as an external test cohort.

Inclusion criteria were as follows: (a) medical information is complete; (b) PCa confirmation through radical surgery or puncture biopsy; (c) BM status ascertained using bone scan or PET-CT (13); (d) Image scanning time and diagnostic time not exceeding two weeks. Exclusion criteria were: (a) Metastasis from other tumor sites; (b) Undergoing surgical or hormonal therapy; (c) poor image quality.

Patient age and clinical symptoms were acquired from medical histories. Clinical data encompassed: (a) Age; (b) PSA levels before intervention therapy; (c) hematuria and Leukocyturia results; (d) GS and ISUP classification; Immunohistochemistry and imaging data. PET-CT or bone scans were employed to distinguish the BM state.

**Figure 1:**
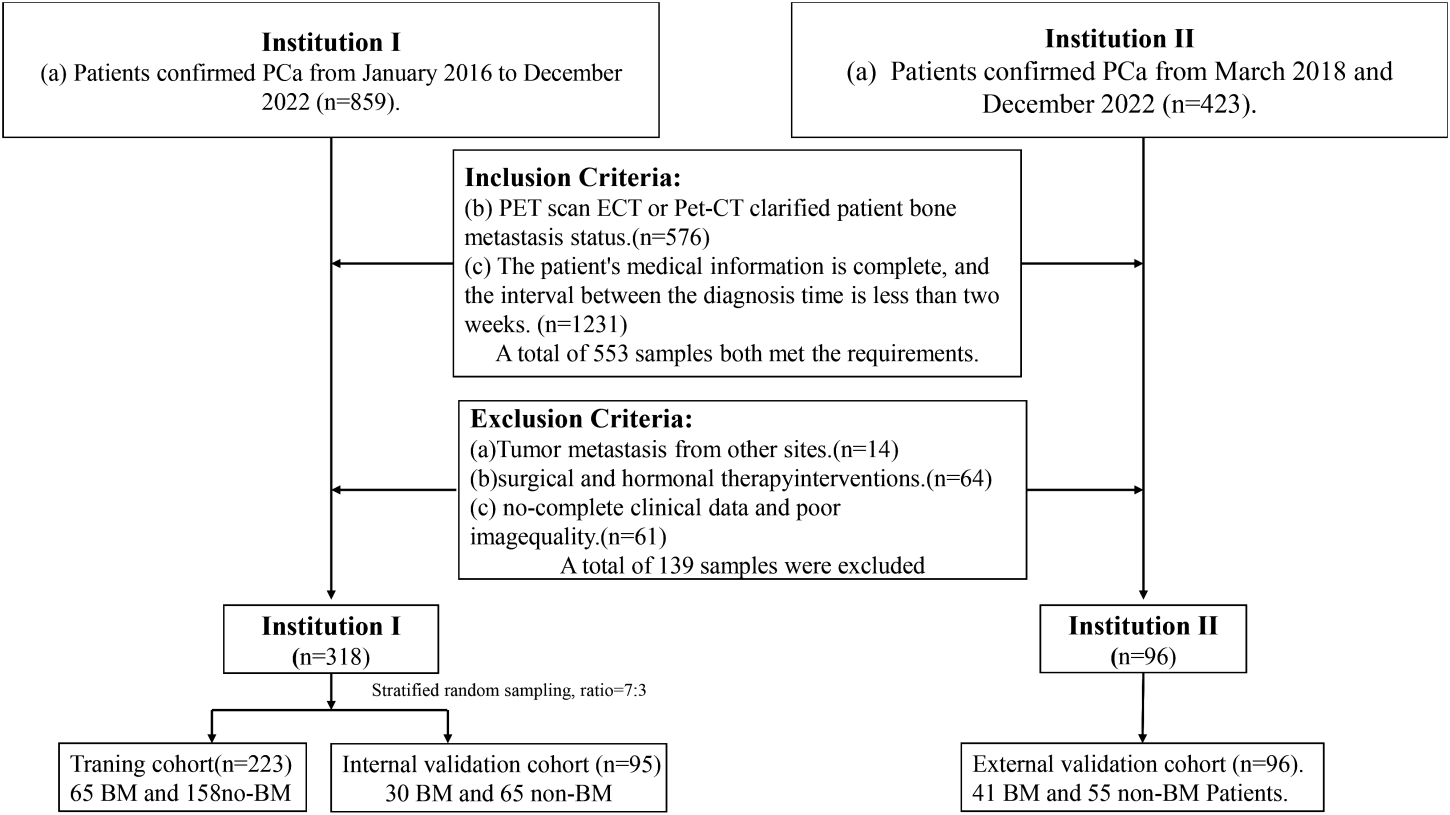
Diagram of the experimental inclusion-exclusion criteria.

Pathology sampling and grading involved sending patients for pathological examination with 3–6 biopsy pieces via rectal ultrasound puncture. GS and ISUP classifications were determined following PCa diagnosis confirmation. The comprehensive screening procedure is illustrated in **Figure 1**.

### 2.2 Experimental equipment

#### 2.2.1 Imaging parameters

Images of patients within the first institution underwent scanning using a 3.0T MRI (750 W 3 T; GE Medical Systems). Using an eight-channel abdominal coil, imaging parameters included: T2-weighted imaging (T2WI) Sequence type: FSE, TR: 2500–5400: msec, TE: 60–120 msec, matrix: 512 × 512, slice thickness: 4 mm, FOV: 20 cm, resolution:0.39 mm/pixel, flip angle: 90°. Diffusion-weighted imaging (DWI): b: 0,800and 1400 s/mm^2^, TR: 5000–6000 msec, TE: 80–120 msec, matrix: 256 × 256, slice thickness: 4 mm, FOV: 20 cm, resolution: 0.78 mm/pixel, flip angle: 90°.

Images of patients within the second institution underwent scanning using a 3.0T MRI system (Siemens Magnetom Verio 3.0T; Siemens Healthcare). With an eight-channel abdominal coil, the imaging parameters were: T2WI Sequence type: TSE, TR: 5000–8000 ms, TE: 80–100 ms,matrix: 320 x 320, slice thickness: 4 mm, FOV: 20 cm, resolution: 0.63 mm/pixel, flip angle: 150°. DWI imaging: b: 0,800 and 1400 s/mm^2^, TR: 3000–5500 ms, TE: 55–85 ms, matrix: 256 x 256, slice thickness: 4 mm, FOV: 20 cm, resolution:0.78 mm/pixel, flip angle: 180°.

### 2.3 Experimental methods

#### 2.3.1 Data processing and augmentation

Prior to the extraction of radiomics features, T2-weighted imaging (T2WI) and diffusion-weighted imaging (DWI) were normalized using the Z-score method. Image outliers were removed by applying the 3σ method (36). The tumor’s region of interest (ROI) on DWI (b: 1400 s/mm2) and T2WI axial images was manually delineated layer by layer by a radiologist with three years of experience, using the 3Dslicer software (version 5.0.2). This delineation was conducted without prior knowledge of the study’s objectives or the BM status, under the supervision of a senior radiologist with 15 years of experience. The ROI for the Apparent Diffusion Coefficient (ADC) sequence was sourced directly from the DWI region.

For the development of auto-sketch models, all images were normalized to a resolution of 224x224x64 using linear interpolation methods, employing the SimpleITK package in Python (version 3.6.2). Due to the different tumor sizes, the ROI images of the tumor regions in the T2WI and DWI sequences were resampled to a resolution of 64x64x32 using the difference method. Pixel consistency was ensured for training the ResNet tumor classification model. Due to the limited patient sample size, the number of images was increased by using data enhancement strategies. These included random rotations of 90, 180, and 270 degrees around the Z-axis of the image pixels and random intensity scaling and offsetting.

#### 2.3.2 Automatic tumor outline model

UNETR model architecture was used to accurately identify regions impacted by Prostate Cancer (PCa). As the top-performing medical classification model of 2021 (37), UNETR model was trained on image from Center 1 and validated by Center 2 image. DWI, T2WI, and ADC images were trained through the three-channel model structure, and Dice similarity coefficient (DSC) was used to evaluate the model prediction efficiency. To optimize our training, we implemented a learning rate scheduler and early stopping criteria, which reduced the learning rate based on validation accuracy and halted training when validation accuracy had not improved for 10 epochs, respectively.

#### 2.3.3 BM prediction model

**Figure 2:**
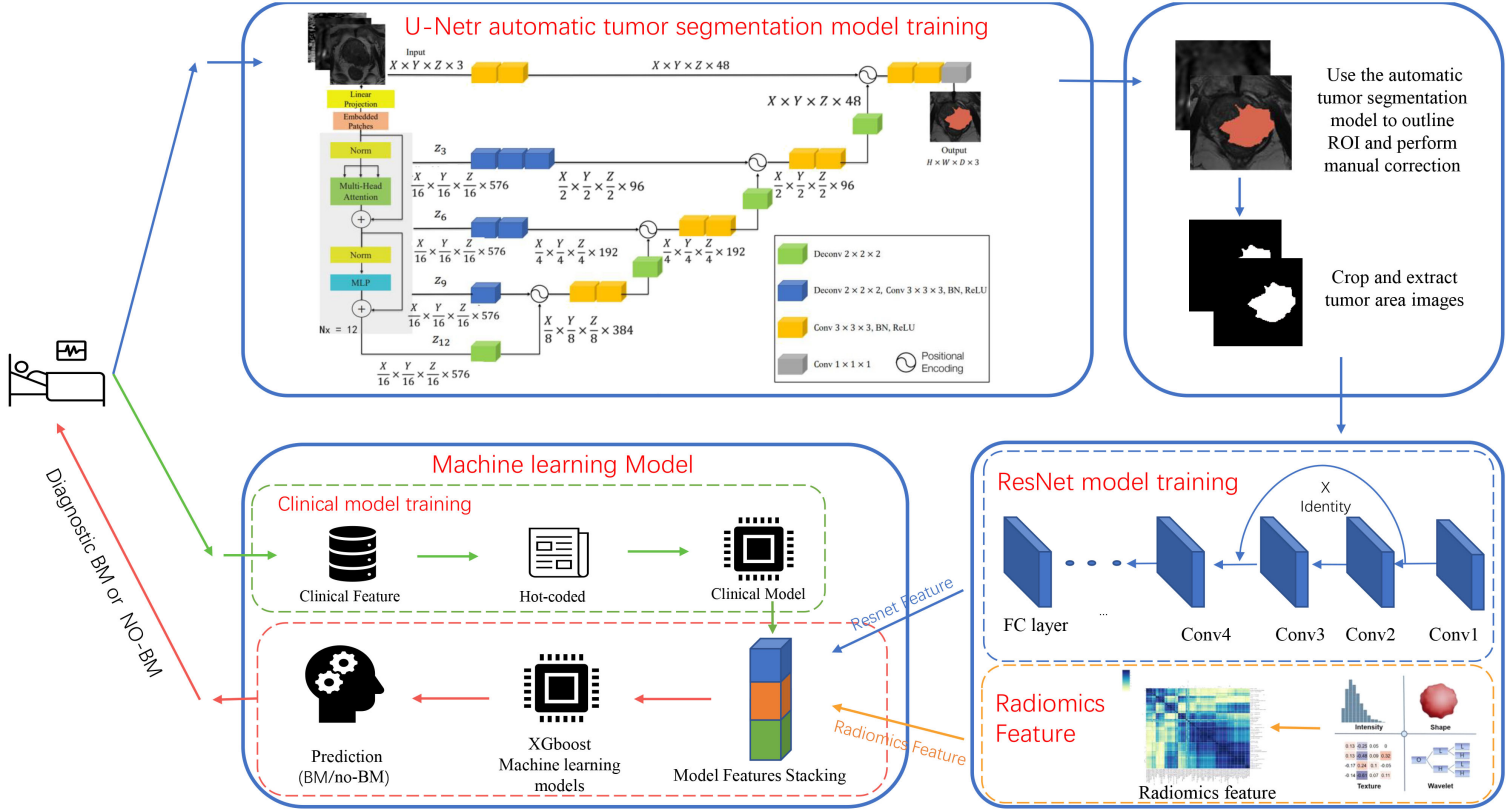
Diagram of the deep learning model architecture. The flowchart shows the experimental design and the specific architecture of the deep learning model.

Radiomics features were extracted from T2WI, DWI, and ADC sequences using pyradiomics. These features were then combined using the XGboost algorithm to create radiomics models. In choosing the structure of a DL model, four different 3D-ResNet network structures, three learning rates, and five optimizers were validated to identify the most effective DL model for predicting BM status. The experimental results can be found in the **Supplementary Document**. Tumor region images were obtained from the UNETR model and then were used to train the ResNet model after radiologist supervision and image enhancement. The validation cohort ROI was utilized to update network parameters, and the internal test cohort was used to assess the model’s performance. The softmax function was employed to produce predictions from the ResNet model. The model with the highest accuracy in the internal test cohort was used to train stacking model (referenced in **Supplementary Tables 1**, **2**). DL features, obtained from the last fully connected layer of all samples, were used to establish a composite model. Finally, The XGboost algorithm was utilized to construct a composite ResNet model (ResNet-C) by integrating the clinical, radiomics, and DL features of the training cohort and validation cohort (**Figure 2**). The model efficiency was subsequently validated through internal and external test cohorts.

#### 2.3.4 Statistical analysis

R (version 4.1.3) and Python (version 3.6.2) were used to conduct statistical analysis and construct models. The Kolmogorov-Smirnov KS test was used to assess the distribution of continuous variables. T-test and Mann-Whitney U test (version 1.7.0)and multi-factor logistic regression analysis were performed to compare the differences between two cohorts. The adjusted p-value was calculated by the Benjamini-Hochberg correction, and Chi-square test was used to analyze the categorical variables. The 95% confidence interval (CI) of the AUC was determined using the bootstrapping method (1000 intervals). From these selected clinical features, we constructed a predictive model using the XGboost algorithm. The receiver operating characteristic (ROC) curve was used to visually demonstrate the prediction ability of ResNet, Radiomics, and ResNet-C models. Delong test was to validate and distinguish differences between the models.

## 3 Results

### 3.1 Clinical model

In our cohort, a mean age of 71.4 years was observed in BM patients, marginally higher than the 70.9 years recorded for the non-BM group; however, this difference was not statistically significant (P=0.687). Additionally, a pronounced 78.3% of BM patients were noted to have Gleason scores of ≥8, in contrast to 46.8% in the non-BM counterparts. Notably, PSA levels exceeding 100 ng/ml were found in 44.1% of BM patients, compared to only 13.5% in the non-BM group. A significant difference in PSA levels between BM and non-BM groups was observed. No significant difference in hematuria and urine leukocyte levels was found between the groups. Through univariate and multivariate analyses, the GS score, ISUP score, and PSA were identified as high-risk factors for predicting PCa’s BM.

### 3.2 Model performance

#### 3.2.1 PCa segmentation model

**Figure 3:**
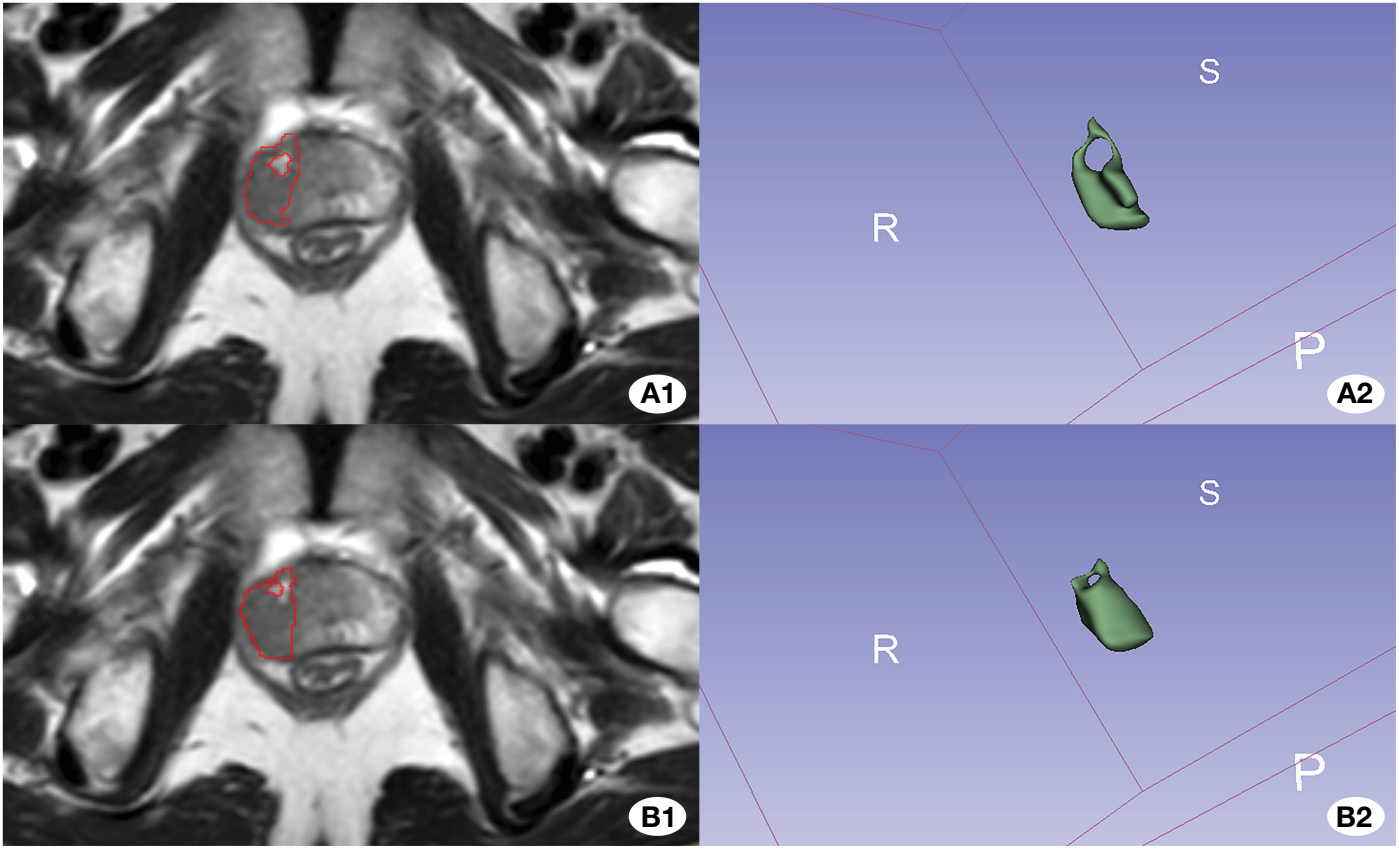
Illustration of the worse effective automatic delineation. In this figure, **(A1)** represents the manual delineation region, while **(A2)** showcases its 3D shape. **(B1)** denotes the automatically recognized region, and **(B2)** displays its 3D form. Notably, the automatic delineation model struggled to accurately identify and circumvent the bleeding foci.

**Figure 4:**
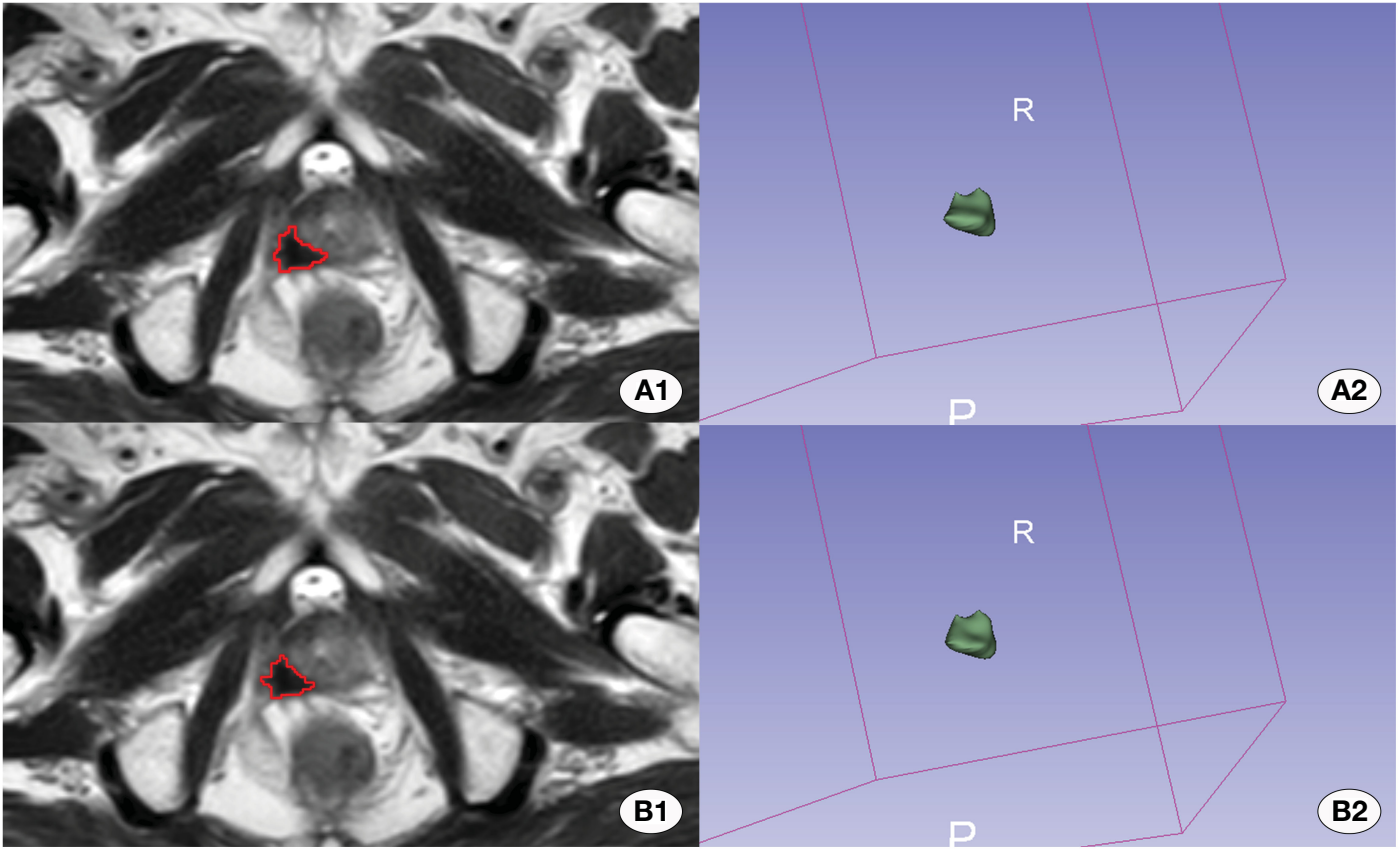
Depiction of the better effective automatic delineation. Here, **(A1)** signifies the manual delineation region, and **(A2)** presents its 3D shape. **(B1)** highlights the automatically recognized region, and **(B2)** reveals its 3D configuration. The automatic delineation model adeptly identified the tumor region in this instance.

The model progressively converged with the increase in training epochs during the training process. The peak accuracy was achieved at epoch 331, recording DSC of 0.607. **Figures 3** and **4** display the High-performing and low-performing samples of the model, respectively.

### 3.3 BM predictive model performance

In the clinical model, an accuracy (ACC) of 0.650 (95% CI 0.558–0.742) was observed in the internal test cohort and 0.668 (95% CI 0.584–0.782) in the external test cohort. Area Under the Curve (AUC) values of 0.713 (95% CI 0.641–0.785) and 0.757 (95% CI 0.661–0.853) were recorded for these cohorts, respectively. For the Radiomics model, an ACC of 0.857 (95% CI 0.796–0.918) was achieved in the internal test cohort and 0.819 (95% CI 0.773–0.865) in the external test cohort. The AUC values were 0.899 (95% CI 0.852–0.946) and 0.852 (95% CI 0.791–0.913) for the internal and external test cohorts, respectively.

Our model was trained using data from the training cohort, and its predictive performance was evaluated with the internal validation cohort. To determine the most effective baseline model, 48 combinations were explored (detailed results are available in the **Supplementary Material**). In the internal validation cohort, ResNet 101 (Adam, LR=0.001) emerged as the top performer, achieving an ACC of 0.875 (95% CI 0.818–0.932) for the internal test cohort and 0.833 (95% CI 0.791–0.875) for the external test cohort. Corresponding AUC values of 0.907 (95% CI 0.860–0.954) and 0.862 (95% CI 0.799–0.925) were recorded.

**Figure 5:**
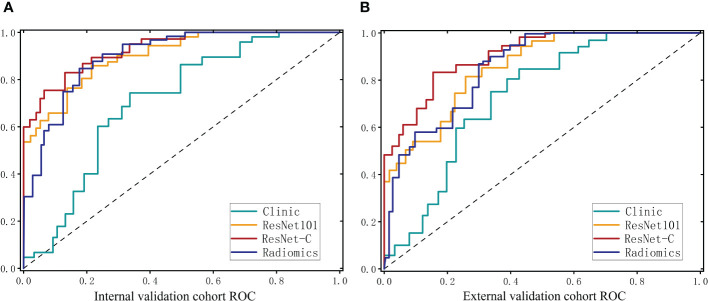
ROC curves for the clinical model. The deep learning model and the integrated model in the internal test cohort **(A)** and the external test cohort **(B)**.

The clinical feature model was then combined with ResNet 101 and radiomics feature to develop the ResNet-C model which model posted an ACC of 0.902 (95% CI 0.867–0.937) in the internal test cohort and 0.885 (95% CI 0.832–0.938) in the external test cohort. The AUC values were 0.934 (95% CI 0.906–0.963) for the internal and 0.903 (95% CI 0.864–0.942) for the external test cohorts, respectively. In the final analysis, Delong validation was used to compare the clinical, ResNet 101, radiomics, and ResNet-C models. The results indicated that the diagnostic efficacy of the Clinic model differs significantly from the other models (refer to **Figure 5**, **Table 1**).

**Table 1:** Predictive model performance effectiveness.

Model	Accuracy		AUC		Delong test*	
	Internal test cohort	External test cohort	Internal test cohort	External test cohort	Internal test cohort	External test cohort
ResNet-C	0.902 (0.867,0.937)	0.885 (0.832,0.938)	0.934 (0.906,0.963)	0.903 (0.864,0.942)	0.006	0.010
ResNet101	0.875 (0.818,0.932)	0.833 (0.791,0.875)	0.907 (0.860,0.954)	0.862 (0.799,0.925)	0.011	0.015
Radiomics	0.852 (0.791,0.913)	0.813 (0.767,0.859)	0.899 (0.852,0.946)	0.852 (0.791,0.913)	0.024	0.035
Clinic	0.650 (0.558,0.742)	0.668 (0.584,0.782)	0.713 (0.641,0.785)	0.757 (0.661,0.853)	–	–

*DeLong test is performed with Clinic as the benchmark, and the 95% confidence interval is listed for AUC and ACC, respectively.

## 4 Discussion

Accurate assessment of BM status is crucial, as the status of BM is a decisive factor for undertaking radical prostatectomy and a key determinant affecting patient prognosis. However, conducting PET-CT/bone scan examinations for all newly diagnosed PCa patients is impractical. Therefore, the development of a convenient and accurate method to identify BM status and provide newly diagnosed patients with precise, personalized BM prediction models is a key objective in current clinical research. This aims to prevent patients eligible for surgical intervention from missing their opportunity for surgery and to assist patients with BM in receiving timely anti-metastasis treatment for their benefit. Recent studies in radiomics and DL suggest that integrated learning with multimodal MRI can facilitate the assessment of a patient’s tumor metastatic status (31–33). Nonetheless, previous studies rarely reported on predicting BM status in PCa using radiomics and DL methods. In our study, to meet the demands of individualized treatment, we developed a more precise model based on bp-MRI scanning strategy. Our stacked model differentiated BM status in internal and external test cohorts with an average AUC value of 0.903–0.934. Additionally, this scanning strategy, reducing contrast agent involvement, is also applicable to patients with contraindications to contrast agents and those with hepatic or renal insufficiency. In this study, we also sought an end-to-end approach to simplify the application of the model in terms of time and manpower costs.

The BM proportions in newly diagnosed PCa patients in our study was 32.8%, higher than the 10% reported in related studies (38). This is due to the fact that patients undergoing PET-CT and bone scan examinations were predominantly high-risk or suspected of having a metastatic status, with inclusion criteria leading to a higher BM detection rate. However, this distortion in BM proportions does not impact the quality of MRI imaging, which is critical for establishing our bp-MRI-based predictive model. Currently, PSA levels are widely used in clinical practice to screen patients at high risk for BM, with some studies indicating a higher risk of BM in populations with high PSA (12–15, 19). Therefore, our study also considers the research value of related indicators. Univariate and multivariate logistic regression analyses showed significant statistical differences in GS score, PSA, and ISUP grading (P < 0.05, **Table 2**), consistent with previous research. However, modeling analysis of clinical features did not provide optimum accuracy. This limitation may be attributed to the unreliability of PSA and GS scores in measuring tumor behavior, as well as the susceptibility of PSA measurements to external influences such as prostatitis, age, endocrine, or metabolic disorders. Additionally, GS scores calculated based on cytological puncture measurements may lead to significant errors (11).

**Table 2:** Characteristics of patients in the training and test cohort.

	BM	Non-BM	Univariable P	Multivariable analysis	
	(n=136)	(n=278)		Odds ratio	P
Age	71.4^*^	70.9^*^	0.786	NA	0.989
Gleason			<0.001	0.348	0.004
6	2 (1.4%)	60 (21.6%)			
7	28 (20.6%)	90 (32.4%)			
≥8	106 (77.9%)	128 (46.0%)			
PSA	169.59^*^	55.16^*^	<0.001	1.003	0.005
<10	17 (12.5%)	47 (16.9%)			
10–100	59 (43.4%)	194 (69.8%)			
>100	60 (44.1%)	37 (13.3%)			
ISUP			<0.001	4.45	<0.001
≤3	30 (22.0%)	150 (54.0%)			
4	45 (33.1%)	65 (23.4%)			
5	61 (44.9%)	63 (22.7%)			
Urine					
hematuria	39 (28.6%)	71 (25.5%)	0.498	NA	0.060
Leukocyt-uria	54 (39.7%)	93 (33.6%)	0.212	NA	0.859

P-value is derived from the univariable association analyses between the clinicopathologic variables and Bone status. The data marked with * are averaged.

Radiomics research based on high-throughput data and the advancements in convolutional neural network-based DL have provided new insights into accurately predicting the biological behavior of tumors (39). Traditional radiomics methods have achieved significant success in this field. Radiomics offers the potential for non-invasive prediction of tumor biological behavior, Wang and Zhang, among others (18, 19), proposed a DCE-MRI BM prediction model using radiomics, achieving an impressive AUC of 0.91. Compared to previous studies, our research has several notable advantages: the imaging strategy of bpMRI limits the utility of existing models. In contrast, our model, based on the bp-MRI scanning strategy, did not perform DCE sequence feature analysis, expanding the model’s audience. Additionally, previous studies did not consider the abstract feature information offered by convolutional neural networks. Deep neural networks can capture nonlinear, interrelated spatial structural features within the target area through convolution and pooling, as well as analyze relationships between distant pixels, all of which are key to accurately predicting BM. The AUC of the ResNet 101 model was 0.907 and 0.862. Thirdly, we conducted ensemble learning of radiomics and DL features, establishing a combined prediction model, ResNet-C, using the XGboost algorithm. The AUCs for Centers 1 and 2 were 0.934 and 0.903, respectively. Machine learning algorithms, compared to linear models, can accurately process nonlinear features. Lastly, our study underwent multicenter validation, providing higher reliability. The DeLong test (all p < 0.05) indicated that MRI image models and clinical risk models have different diagnostic efficacies, proving the necessity of stacking different modal models.

**Figure 6:**
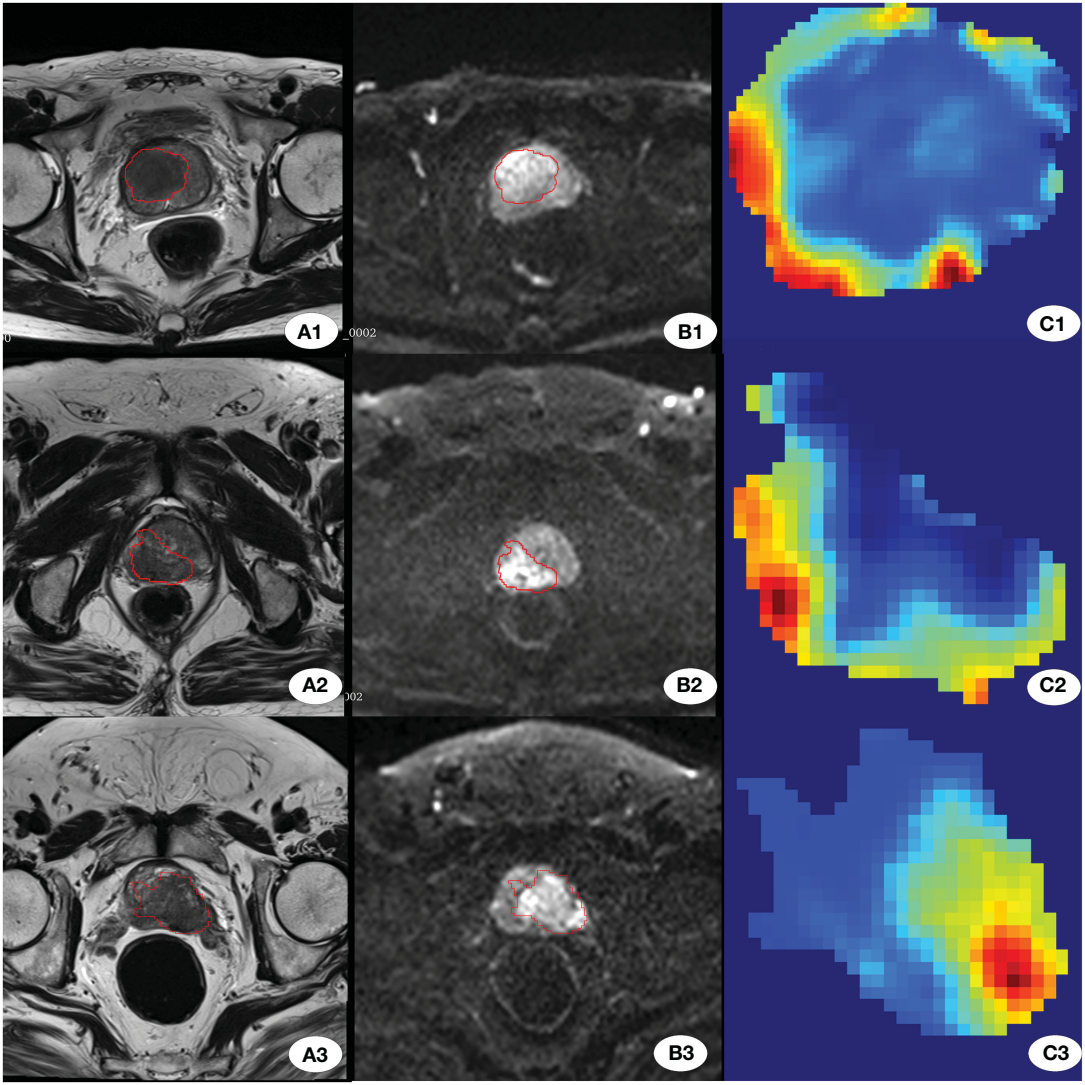
Grad_GAM. **(A1-A3)** show T2-weighted images from three different patients with BM. **(B1-B3)** display the corresponding DWI images from the same three patients. **(C1-C3)** present the Grad-GAM images of the largest cross-sectional area of the tumor for each patient.

Radiomics and DL have achieved encouraging results, but the analysis of tumor regions largely depends on manual delineation. Extracting tumor information from MRI multiple sequences and multiple planes using manual methods is a time-consuming task. Accurate segmentation of images aids in advancing the application of radiomic models in clinical settings. Moreover, the automatic recognition of tumor regions is a crucial component of an end-to-end model. Therefore, this study has made some attempts in this area. After validation with an external test cohort, our established tumor segmentation model yielded a DSC value of 0.607. This indicates that the model can only be semi-automated; while it can more accurately locate tumors, the precise determination of tumor extent and boundaries still requires manual intervention by radiologists. Our initial goal was full automation, but the model struggles to differentiate PCa from other abnormalities (such as inflammation and hemorrhage), challenges that may stem from overlapping signal characteristics between lesions and PCa. To ensure accuracy in tumor regions, radiologists must also examine these differences. Deep learning networks are commonly referred to as “black box” structures due to their complex and often non-transparent nature. Therefore the Grad-CAM technique is employed to attempt to uncover and interpret the decision-making process of convolutional neural networks. This is achieved through the analysis of the feature weight maps of the final convolutional layer. In observing the ResNet model, it was noted that the decision-making process relies on the tumor margin areas (**Figure 6**). We posit that this reliance may be attributed to the interactions between the microenvironment of the active tumor cells at the periphery and the surrounding normal tissue (40).

The results of our study’s model, validated through internal and external verification sets, demonstrate significant potential. However, the establishment of end-to-end models also presents numerous challenges. Concerns regarding data privacy and security, coupled with insufficient interpretability leading to difficulties for physicians in understanding the model’s decision-making process, pose substantial obstacles. Additionally, the validation and adaptability testing in real clinical settings demands considerable time and resources. Furthermore, issues related to regulatory compliance, physician acceptance, and model usability are critical. Successful integration of the medical end-to-end model into clinical practice necessitates interdisciplinary collaboration and continuous improvement of model performance. The visualization application of the model requires the design of a software platform, and we are actively collaborating with software companies to seamlessly integrate the model into software modules embedded in the medical record diagnostic system, ultimately benefiting patients.

Despite promising findings, our study has limitations. Firstly, the DL and radiomics models were established using retrospective data. Prospective data from more clinical trials in the future will improve the clinical evidence of our models. Secondly, differences in examination equipment might affect the model’s performance. In the external test cohort, a deeper network model, ResNet152, showed better diagnostic performance (see **Supplementary Tables 1**, **2**), We believe this is attributed to deeper model architectures, which aid in better understanding the inherent relationships between different regions of the tumor and assist the model in extracting more discriminative abstract features from the imaging data, necessitating an expansion of the number of centers in future research to confirm this phenomenon. Additionally, our study neglected 1.5T images, due to the challenge of accurately delineating tumor regions on T1 sequence images, the exploration of the value of T1 sequence images has been omitted in this study. Due to the limited sample size, this study analyzed images of delineated tumor regions. However, this introduces a drawback: normalizing tumor sizes during training prevents our model from assessing the correlation between tumor size and bone metastasis. In fact, with a sufficiently large training set, CNNs may not require highly accurate tumor masks as input. Therefore, our future research goal is to broaden the sample scope, and increase its scale and diversity, thus further enhancing the performance of our model.

In summary, we have developed an integrated learning model using bp-MRI images to accurately predict the BM status of PCa patients. For PCa patients, this model can assist urologists in deciding whether there is an opportunity for radical surgery, which is positively significant for the patient’s treatment approach and prognosis.

## 5 Conclusions

Our study combined clinical parameters with prostate bpMRI, offering clinicians a non-invasive tool to inform treatment decisions for prostate patients. Additionally, we developed an automatic tumor delineation model to streamline the process and augment efficiency, increasing its potential for clinical adoption.

## Data availability statement

The original contributions presented in the study are included in the article/**Supplementary Material**. Further inquiries can be directed to the corresponding authors.

## Ethics statement

The studies involving humans were approved by Ethics committees of Xiangyang First People’s Hospital (approval number: XYYYE20210020) and Xiangyang Central Hospital (approval number: 2022–020-01). The studies were conducted in accordance with the local legislation and institutional requirements. The ethicscommittee/institutional review board waived the requirement of written informed consent for participation from the participants or the participants’ legal guardians/next of kin because this was a retrospective study.

## Author contributions

SX: Writing – review & editing, Writing – original draft, Visualization, Software, Resources, Project administration, Methodology, Funding acquisition, Data curation, Conceptualization. ST: Writing – review & editing, Writing – original draft, Visualization, Supervision, Software, Resources, Methodology, Investigation, Data curation. HX: Writing – review & editing, Validation, Methodology, Investigation, Data curation. ZS: Writing – review & editing, Validation, Supervision, Methodology, Investigation. WY: Writing – review & editing, Supervision, Investigation, Data curation. DM: Writing – review & editing, Validation, Project administration, Investigation. XT: Writing – review & editing, Supervision, Software, Resources, Methodology, Funding acquisition, Formal analysis, Data curation, Conceptualization. ZJ: Writing – review & editing, Visualization, Supervision, Resources, Methodology, Funding acquisition, Data curation, Conceptualization. YF: Writing – review & editing, Validation, Supervision, Resources, Methodology, Funding acquisition, Formal analysis, Conceptualization.
