# Supplementary material for: A semi-automatic deep learning model based on biparametric MRI scanning strategy to predict bone metastases in newly diagnosed prostate cancer patients
Source: Front Oncol. 2024 Jun 11;14:1298516. doi: 10.3389/fonc.2024.1298516 (PMC11196796; doi:10.3389/fonc.2024.1298516)
Supplement: Supplementary file 1 [file DataSheet_1.docx]

Supplementary Material

# U-NETR technical details

We implemented the U-NETR model for tumor outlining in the PyTorch environment using the RTX3090TI graphics card. This model, based on the U-NETR architecture, follows a contracting-expanding pattern. The encoder, composed of a series of transformers, is linked to the decoder through skip connections. We project the patches into a consistent K-dimensional embedding space using a linear layer. This is followed by a sequence of transformer blocks, each containing multi-head self-attention (MSA) and multi-layer perceptron (MLP) sublayers. Here, the Norm module denotes data normalization, while the MLP is made up of two linear layers using GELU as the activation function.

At the encoder's bottleneck (specifically, the last layer of the inverter), a deconvolution layer increases the transformed feature map's resolution by a factor of 2. In the decoder, consistent with the Unet structure, the feature map from the previous transformer output (like z9) is processed by sequential 3x3x3 convolutional layers. The output is then upsampled via a deconvolutional layer. This approach is applied iteratively to all subsequent layers, maintaining the original input resolution. The final step involves passing the result through a 1x1x1 convolutional layer with a softmax activation.

The training was conducted using the Adam optimizer, set to an initial learning rate of 0.001, across 800 epochs. The network featured L = 12 layers with an embedding size of k = 384. Patches of 16x16x16 were utilized. For efficient model training, we adopted a sliding window strategy with an 80% overlap between adjacent patches. Considering the limited scale of medical imaging cases, we applied a data augmentation technique during training. This involved random rotations of image pixels by 90, 180, and 270 degrees, combined with random scaling and intensity offset. This method generated a total of 2862 sample sets. Our model was engineered to accept three channels of input data corresponding to ADC, T2WI, and DWI. We initially trained 3D images by transforming them into spatial vectors of specified resolution and then integrated them into our model. This integration is expressed as: length (H) x width (W) x height (D) x number of modes (C). The decoder is based on a traditional U-Net design with a 3x3x3 convolutional kernel size and a 2x2x2 pooling layer.

# Resnet 3D model technical details

We utilized the RTX3090TI graphics card in the PyTorch environment (https://pytorch.org/) to train the ResNet 3D model. Our experimental sample data were saved in the nii.gz format. Given the scarcity of 3D image-based classification models in the medical realm, we tested various optimizers including SGD, Adadelta, Adagrad, and Adam, with different learning rates (0.01, 0.001, and 0.0005). All data underwent the previously mentioned augmentation process to enhance model stability. The validation set image is used to determine the optimal model parameters. The batch size = 32. During training, L2 regularization and early stopping are used to prevent overfitting were used to fine-tune optimization, and to stop training when validation accuracy had not improved for 10 epochs. Loss ratio is used to evaluate the model performance. Simultaneously, we expanded the model's channels (C) to three to accommodate the MRI multi-sequence application environment. This configuration is represented as: length (H) x width (W) x height (D) x number of modes (C). A convolutional kernel size of 3x3x3 was used, and a pooling layer of 2x2x2 was adopted.

# XGboost model details.

eXtreme GradientBoosting (XGboost) is the learning rate = 0.13, gamma = 0.4, maximum tree depth = 4, subsample = 0.8, alpha = 1e-05, lambda = 1,scale_pos_weight = 1, and the number of rounds = 643.

**Supplementary Table1**. TOP10.Test cohort model performance.

| Rank | Model | Learn Rate | Optimizer | Accuracy | AUC |
| --- | --- | --- | --- | --- | --- |
| 1 | ResNet-C | - | - | 0.902 | 0.935 |
| 2 | ResNet101 | 0.001 | Adam | 0.853 | 0.907 |
| 3 | ResNet101 | 0.001 | SGD | 0.849 | 0.900 |
| 4 | ResNet152 | 0.001 | Adam | 0.849 | 0.898 |
| 5 | ResNet101 | 0.01 | Adam | 0.846 | 0.892 |
| 6 | ResNet50 | 0.01 | Adam | 0.846 | 0.896 |
| 7 | ResNet101 | 0.0005 | SGD | 0.846 | 0.878 |
| 8 | ResNet18 | 0.0005 | Adam | 0.843 | 0.886 |
| 9 | ResNet101 | 0.0005 | Adagrad | 0.840 | 0.882 |
| 10 | ResNet50 | 0.01 | SGD | 0.837 | 0.875 |
| … | … | … | … | … | … |
| 49 | Clinic | - | - | 0.700 | 0.773 |

**Supplementary Table** **2**. TOP10.External test cohort model performance

| Rank | Model | Learn Rate | Optimizer | Accuracy | AUC |
| --- | --- | --- | --- | --- | --- |
| 1 | ResNet-C | - | - | 0.885 | 0.903 |
| 2 | ResNet152 | 0.001 | Adam | 0.833 | 0.874 |
| 3 | ResNet101 | 0.001 | SGD | 0.823 | 0.865 |
| 4 | ResNet101 | 0.001 | Adam | 0.823 | 0.862 |
| 5 | ResNet50 | 0.01 | Adam | 0.813 | 0.857 |
| 6 | ResNet101 | 0.0005 | SGD | 0.813 | 0.853 |
| 7 | ResNet50 | 0.01 | Adam | 0.813 | 0.848 |
| 8 | ResNet152 | 0.01 | Adagrad | 0.813 | 0.851 |
| 9 | ResNet18 | 0.01 | Adam | 0.802 | 0.839 |
| 10 | ResNet101 | 0.01 | SGD | 0.802 | 0.842 |
| … | … | … | … | … | … |
| 49 | Clinic | - | - | 0.688 | 0.747 |
